# Gout—Cold-Water Treatment—Frequent Relapse—Translation to the Head and Stomach—Ultimate Recovery

**Published:** 1844-02-10

**Authors:** 


GOUT—COLD-WATER TREATMENT—TRANSLATION TO THE HEART AND STOMACH—DEATH.

GOUT—COLD-WATER TREATMENT—FREQUENT RELAPSE—TRANSLATION TO THE HEAD AND STOMACH—ULTIMATE RECOVERY.

A gentleman, about forty years of age, who had been subject to severe fits of gout for many years, complained of general indisposition, and had one ancle inflamed, swollen, and exquisitely painful; cold applications were ordered externally, and a cordial internally. Next day the ancle was relieved, but the knees had become painful: the same application directed to the knees. On the following day there was severe pain in the head, and great uneasiness in the stomach, attended with nausea and flatulence; a draught with æther and laudanum was ordered him. The succeeding day he was better in every respect, and in a few days was quite well. He remained so for twelve months, had then a similar attack, and, after being treated in the same manner, was again able in a few days to pursue his usual avocations; but in less than a month had a return of the gouty symptoms. His own opinion was that the gout had lurked in his system ever since the last attack; at the same time, he felt convinced that though the application of cold might not eradicate the disorder, it yet afforded him so much relief as to induce him to wish that it should again be tried. This was done, and in six days nothing, apparently, but weakness remained. Four days afterwards, however, the left ancle (not the one which had suffered last), became inflamed and painful; the patient had immediate recourse to his favorite remedy, but had no sooner applied the first wet cloth, than he felt an unusual torpor pervade his whole frame. He suffered intense pain in the stomach, attended with frequent vomiting; the head was much affected, and he was at intervals delirious. He took hot brandy and water, and heated wine with spice, which afforded him some relief; at the same time the cold, which had been used for about two hours, was discontinued. On the following day he was better, though very languid; the inflammation did not return in the left ancle. Next day he continued tolerably well, but on the day after, a more violent attack than any of the former took place in the right hand. Warm applications were now employed. The stomach, head, and constitution generally, being as much affected as before, and on the symptoms of internal disorder having abated, the local affection of the hand continuing, cold was again applied, and the patient recovered.—*Prov. Med. Jour.* Oct. 21, 1842.

